# Functional Parameters of Hybridoma Cells: Methods of Evaluation and Biotechnological Relevance

**DOI:** 10.3390/antib15040067

**Published:** 2026-08-04

**Authors:** Tatiana Bezukladnikova, Svetlana Zamorina, Sergey Lazarev, Mikhail Rayev

**Affiliations:** Perm Federal Research Center of the Ural Branch of the Russian Academy of Sciences, Institute of Ecology and Genetics of Microorganisms, 614081 Perm, Russialasest1999@gmail.com (S.L.)

**Keywords:** hybridoma technology, viability, proliferation, productivity, monoclonal antibody, functional parameters

## Abstract

Hybridoma technology remains one of the most reliable and widely used platforms for generating highly specific monoclonal antibodies for use in diagnostics, fundamental research, and clinical practice. Moreover, the combination of unlimited proliferative capacity with the preservation of a key B-lymphocyte function, antibody production, enables hybridoma cells to support a comprehensive functional evaluation of cellular responses. Thus, by integrating analysis of proliferation, viability, and productivity, hybridoma-based approaches enable the detection of differential modulatory effects and offer a nuanced assessment of compound bioactivity. This review provides a comprehensive analysis of the functional parameters of hybridoma cells: viability, proliferation, and productivity, as well as the methods used for their evaluation. The main stages of hybridoma cell generation, advances in hybridoma technology, and current applications of hybridoma cells are also reviewed. A key aspect of this review is the differential modulation of functional parameters of hybridoma cells. Modulation of culture conditions and bioactive compounds can differentially influence growth dynamics and specific antibody yield, often revealing an inverse relationship between proliferation and productivity.

## 1. Introduction

The modern pharmaceutical, biomedical, and biotechnology industries are characterized by a continual increase in the number of synthesized compounds, necessitating rapid and reliable evaluation of their biological activity. The early identification of substances with therapeutic potential, as well as the detection of compounds with undesirable toxic or immunomodulatory effects, represents a key objective of preclinical research.

In vitro studies play a central role in the primary screening of biological activity [1,2]. However, most conventional cell-based assays primarily focus on assessing basic parameters such as proliferation and viability [3,4]. Despite their utility, these parameters reflect general cytotoxic effects and do not fully capture changes in specialized cellular functions. In this regard, functional characteristics, especially the secretory activity of immunocompetent cells, are critical for understanding the mechanisms of action of biologically active compounds.

In this context, hybridoma cell lines are particulary important, as they combine the capacity for unlimited proliferation with the preservation of a key specialized function of B lymphocytes—antibody production [5]. This enables hybridoma cells to support a comprehensive functional evaluation of cellular responses, including the analysis of proliferation, viability, and antibody production. This approach enables the identification of differential modulatory effects of compounds, including cases where inhibition of cell growth is followed by enhanced antibody secretion [6].

This review focuses on the functional characteristics of hybridoma cells, including proliferation, viability, and productivity, as well as the methods used for their evaluation. It also summarizes key advances in hybridoma technology and opportunities for its application. Hybridoma cells are considered not only a source of monoclonal antibodies but also as a promising experimental platform for a comprehensive analysis of cellular response. This analysis includes the evaluation of proliferation, viability, and productivity.

A literature search was conducted using PubMed, ScienceDirect, and Google Scholar databases. Articles published up to and including 2026 were considered. The following keywords were used in various combinations: hybridoma, hybridoma technology, viability, proliferation, productivity, apoptosis, monoclonal antibody, cell sorting, cell cloning, production rate, production stimulating.

## 2. Hybridoma Technology, General Information

### 2.1. Key Stages in Hybridoma Generation

Hybridoma technology, originally described by Georges J. F. Köhler and César Milstein in 1975, made it possible to overcome a key challenge: the production of unlimited quantities of antibodies with identical specificity [7]. This breakthrough is widely considered one of the most significant milestones in the field of biotechnology. Hybridoma technology greatly expanded the capacity for both the detection and production of antibodies, thereby laying the foundation for the widespread application of monoclonal antibodies in scientific research, diagnostics, therapeutics, and biotechnology [8].

**Figure 1:**
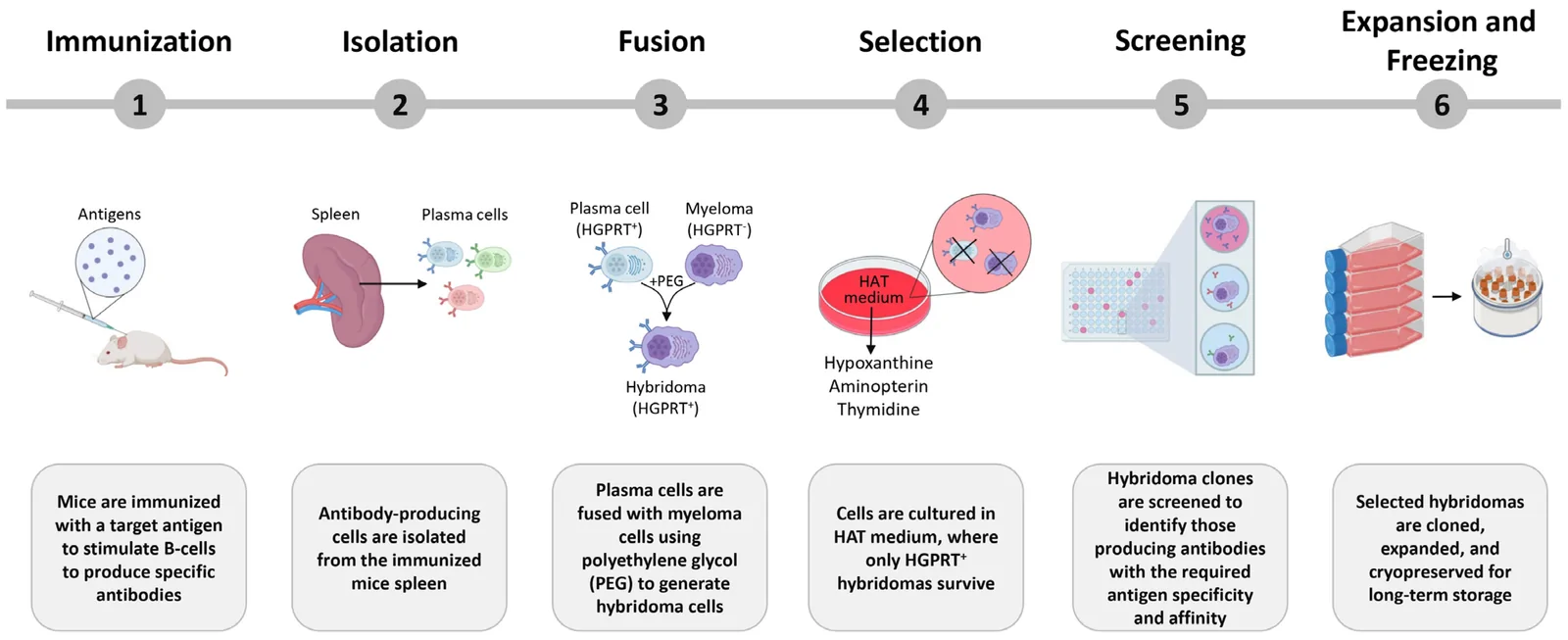
Illustrative overview for the key stages in hybridoma technology (created with BioRender.com).

Classical hybridoma technology is a method based on the fusion of an immortal myeloma cell with a B-lymphocyte that produces monoclonal antibodies of predefined specificity [7]. Thus, hybridoma cell lines feature both stable antibody expression and the ability for continuous proliferation. Generating hybridoma cell lines is a multistep and labor-intensive process, with its key stages shown in Figure 1.

The initial step involves the immunization of laboratory animals, which receive multiple injections of the selected antigen over several weeks to stimulate specific antibody production [9]. Classical murine hybridomas are typically generated using BALB/c mice [10].

During the next step, activated B-lymphocytes are harvested from the spleen of an immunized animal and fused with HGPRT-deficient myeloma cells [5]. Commonly used myeloma partners include P3X63Ag8-U1 [11,12], SP2/0 [12,13,14,15,16], P3U1 [17], P3X63-Ag8.653 [12,18,19,20], and NS1 [13,21]. In practice, non-secreting myeloma lines are preferred. Polyethylene glycol is commonly used to induce cell fusion [7]. Alternatively, viruses such as Sendai virus [7,22] or electrofusion methods [21,23,24,25] may be used. Electrofusion demonstrates significantly higher efficiency than chemical and viral methods, because it offers greater controllability, synchrony, and hybrid yields, with lower toxicity and higher hybrid viability [24,25,26].

After fusion, some intact cells remain in the culture. Hybridoma selection is carried out in HAT medium containing hypoxanthine, aminopterin, and thymidine [7]. Aminopterin inhibits de novo nucleotide synthesis, whereas hypoxanthine and thymidine are utilized in nucleotide production via the salvage pathway in HGPRT-positive cells. Due to their limited in vitro lifespan, unfused B-lymphocytes die within a few days up to two weeks. Myeloma cells that lack functional HGPRT are unable to use the salvage pathway and consequently do not survive. As a result, only hybridoma cells survive in HAT medium; these hybrid cells combine unlimited proliferation capacity inherited from the myeloma partner with the ability to express antibodies of defined specificity derived from B cells [5].

Hybridomas are then cloned by limiting dilution [27] to assess the characteristics of the monoclonal antibodies. In the final stage, selected antibody-secreting clones are expanded and cryopreserved [28] to ensure long-term storage and subsequent application in in vivo and in vitro culture systems [5].

### 2.2. Advances in Hybridoma Technology

Since its introduction over five decades ago, hybridoma technology has been significantly refined in its key stages. Some of these advances are summarized in Table 1.

A fusion stage modification significantly enhanced hybridoma yield. Preincubation of myeloma cells and lymphocytes in the presence of 0.25% PEG for 90 min at 37 °C resulted in an approximately tenfold increase in the proportion of wells containing antibody-secreting hybridomas [29].

Significant progress has been achieved in the field of electrofusion [24,25,26]. Optimization of pulse parameters, including the use of nanosecond and microsecond pulses with low peak power, has enabled a 1.5- to 3-fold increase in fusion efficiency compared with conventional high-power microsecond pulses [30]. Enriching cells using fluorescence-activated cell sorting (FACS) prior to electrofusion has also been shown to improve the yield of functional hybridomas [21]. Furthermore, a microfluidic droplet-based platform for hybridoma generation has been developed [49], along with a microfluidic chip that enables cell electrofusion within microdroplets [31].

In addition to these technical advances, modifications of culture media have been proposed to improve cell survival following fusion, including the addition of macrophage-conditioned medium [32]. Similarly, a conditioned medium derived from cultured rat thymocytes effectively replaces feeder cells and significantly enhances hybridoma growth [29].

FACS has become an important tool not only at the stage of cell preparation for fusion but also in the selection of antigen-specific hybridomas [35]. Early strategies involved labeling cells with antigen-coated fluorescent microspheres to identify antibody-secreting clones [33]. Current methods involve multistep fluorescence detection, including antigen binding followed by secondary reporter antibodies [34], as well as genetic modification of hybridomas to express a biotinylated surface marker that captures secreted antibodies on the surface of the producing cell [36]. Furthermore, membrane immunoglobulin-based screening (MIHS), which utilizes antigen binding to the hybridoma B-cell receptor, has been introduced [37]. Overall, FACS-based methods significantly increase the number of analyzed cells and enable the generation of clonal lines with high reliability [22].

Magnetic particles functionalized with the target antigen allow selective isolation of antigen-specific hybridomas, providing up to a 300-fold enrichment of the desired cell population [38].

Cultivation in semisolid media, such as those based on methylcellulose [39] or agar [40], is one of the most effective approaches for hybridoma clone selection. In liquid culture, the lack of spatial isolation leads to overgrowth of rapidly proliferating, low-producing cells and loss of slower-growing but potentially high-producing clones [50]. Semisolid media overcome this limitation through spatial isolation: each hybridoma forms an individual colony, thereby preventing repeated selection of identical variants and enabling independent evaluation of each clone’s productivity [51]. Due to matrix viscosity, secreted antibodies remain localized around the colony, forming a characteristic “halo” of immune complexes that can be evaluated by fluorescence intensity [51].

Automation of this approach is achieved using the ClonePix 2 platform (formerly Genetic ClonePix FL, Molecular Devices) [41,42]. This platform enables in situ screening of colonies and subsequent isolation of antigen-specific clones of a defined isotype [41]. Moreover, it allows selection of the most productive variants and, in many cases, generates high-producing lines after the first post-fusion cultivation step [22,41]. Integration of ClonePix with FACS further enhances selection efficiency and reduces development time [52].

A promising approach involves the use of self-seeding microwell chips coupled with an activated membrane for capturing antibodies secreted by individual cells [43]. This platform enables high-throughput screening and isolation of up to 6400 clones per day, providing a rapid and scalable solution for early-stage clone selection.

Surface plasmon resonance (SPR) biosensing provides a label-free, real-time platform for kinetic characterization of antibody–antigen interactions directly from hybridoma supernatants [44,45,46,47,53]. In contrast to end-point assays such as ELISA, SPR enables simultaneous determination of association (*k_a_*) and dissociation (*k_d_*) rate constants, and the capture format (using Fc-specific antibodies) avoids avidity artifacts and allows true affinity measurements [44,45,46,47]. The dissociation rate constant has been established as the most reliable parameter for ranking antibody affinity, often identifying high-affinity clones [44,46].

Microarray-based method is also used to screen for high-affinity hybridoma clones. This method is based on an antibody-immobilized format in which raw hybridoma supernatants are spotted onto epoxysilanized glass slides pre-coated with Cys-Protein G, enabling oriented capture of immunoglobulins directly from complex media. Subsequently, a monovalent fluorescent hapten tracer is incubated in parallel noncompetitive and competitive chambers on the same slide, and the ratio of fluorescence signals allows the identification of high-affinity clones [48].

Taken together, these developments have markedly enhanced the efficiency of hybridoma technology, accelerated the generation of monoclonal antibodies, and increased the likelihood of isolating rare, high-producing, antigen-specific clones.

Initially, hybridoma technology was predominantly used for the production of mouse antibodies; however, with further methodological development, it has been widely applied to generate monoclonal antibodies of diverse origin, including those derived from chicken [54], goat [55], rat [56], and rabbit [57]. Rabbit antibodies are of particular interest because the rabbit immune system can generate antibodies with higher affinity and broader molecular recognition, including phosphopeptides, carbohydrates, haptens and antigens that are weakly immunogenic in mice [58,59,60,61,62].

Despite the success of hybridoma technology in generating high-affinity and highly specific antibodies, the therapeutic application of non-human antibodies is limited because they induce immunogenic responses [63] and adverse effects [64], which can reduce treatment efficacy and negatively affect pharmacokinetic properties [65]. To overcome these challenges, approaches for the development of chimeric [66] and humanized monoclonal antibodies [67] have been established. Concurrently, alternative antibody discovery platforms independent of hybridoma technology have been developed, including phage display [68,69], single B-cell–based methods [70], and de novo approaches based on artificial intelligence [71]. These and other modern antibody generation technologies have been extensively reviewed elsewhere [72,73,74,75].

### 2.3. Current Applications of Hybridoma Technology

**Figure 2:**
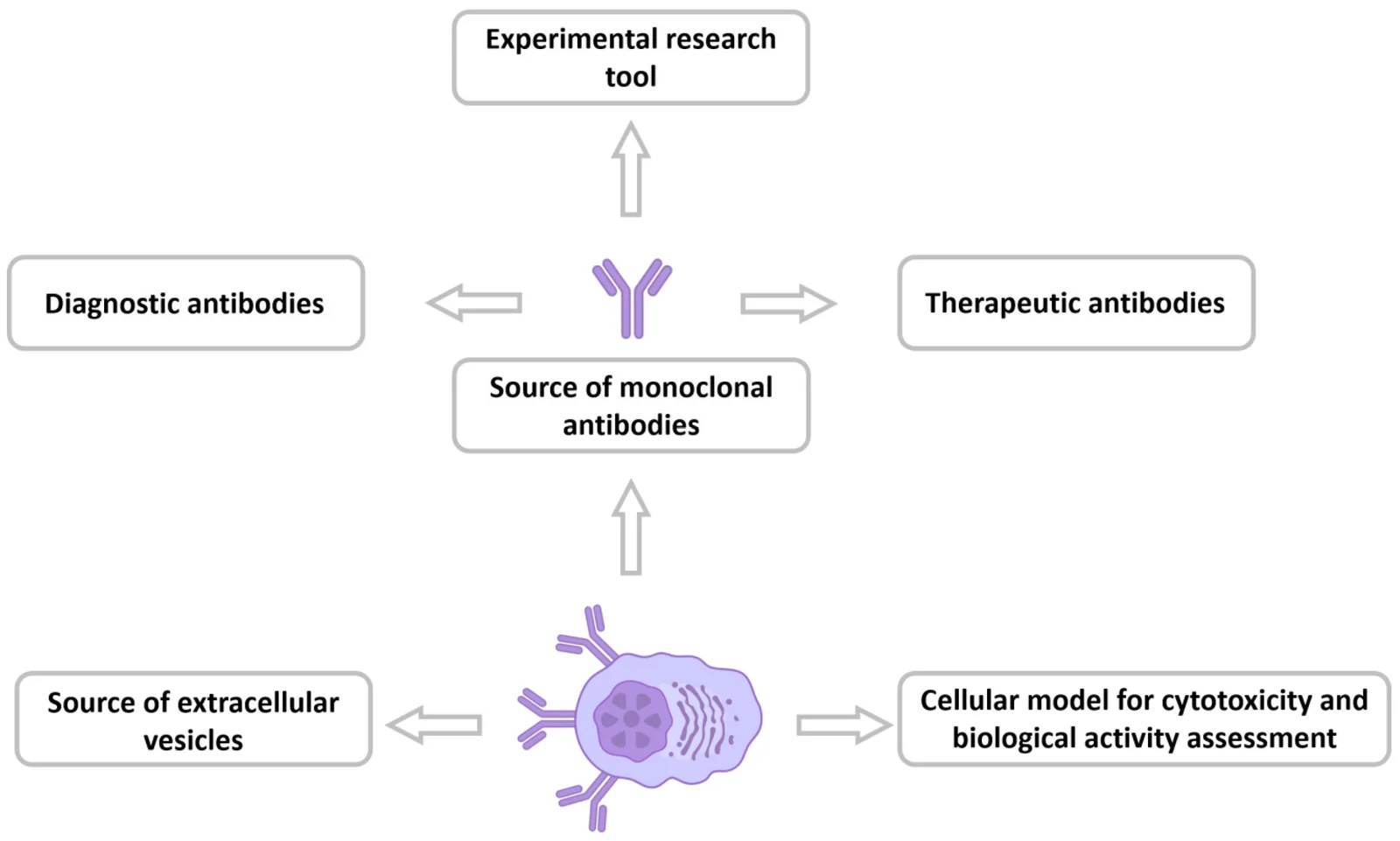
Illustrative overview of hybridoma cell applications and the uses of their monoclonal antibodies (Created with BioRender.com).

Hybridoma technology remains one of the most reliable and widely utilized platforms for monoclonal antibody generation. Its relevance is evidenced by the continuous increase in publications describing the development and application of novel monoclonal antibodies produced using this approach. This technology enables the generation of highly specific antibodies that are extensively used in diagnostics, fundamental research, veterinary medicine, and clinical practice. Figure 2 illustrates the application of hybridoma technology.

Comprehensive coverage of all publications in this field is beyond the scope of this review. Therefore, the literature was selected based on its relevance, scientific novelty, and practical significance, with a focus on recent studies that reflect key directions in the development and application of monoclonal antibodies and hybridoma technology.

Diagnostics represent one of the major application areas of hybridoma-derived antibodies. For example, monoclonal antibodies against the denatured monomeric non-structural protein of Zika virus ZNS1 have been proposed as a basis for highly sensitive and specific indirect competitive ELISAs for early flavivirus detection [76]. Monoclonal antibodies are also used in ELISAs for the detection of calreticulin gene (CALR) mutations associated with essential thrombocythemia and primary myelofibrosis [77]. Chinese researchers have generated the first monoclonal antibodies against giant panda (*Ailuropoda melanoleuca*) vascular endothelial growth factor A (VEGFA), which are considered a novel tool for studying reproductive biology and oncology in this species [14]. Furthermore, monoclonal antibodies against the VP27 protein of goose astrovirus GoAstV-2 have been proposed as promising candidates for both diagnostics and epitope-based vaccine development [78]. Similarly, monoclonal antibodies targeting the VP3 protein of infectious bursal disease virus have enabled the identification of a neutralizing antigenic epitope and provided a basis for designing multiantigen vaccines [15]. In addition, antibodies targeting the SWAP antigen of *Schistosoma mansoni* have demonstrated high performance in schistosomiasis immunodiagnostics, enhancing sensitivity, specificity, and staging accuracy [11].

Environmental analysis and food analysis also represent an area of hybridoma-derived antibodies application. Monoclonal antibodies have been developed for the ELISA-based detection of citrinin in wine, a toxic compound of concern for human health [79]. Similarly, antibodies targeting the natural antioxidant dihydromyricetin have been generated for its detection in *Ampelopsis grossedentata* [80].

Another important research direction is the development of therapeutically and functionally active antibodies. For example, a monoclonal antibody against ITGA4 has been reported to induce cell death in NK/T-cell lymphoma by forming large pores in the cell membrane [17]. Hybridoma-derived neutralizing monoclonal antibodies targeting the beta and delta variants of SARS-CoV-2 are considered promising candidates for therapeutic application after further optimization [81].

Monoclonal antibodies specific to peanut-reactive human IgE passively sensitize effector cells and are widely used in in vitro models of the effector phase of food allergy [82].

Hybridoma cells represent an important source of genetic material for antibody engineering. Genes derived from hybridomas are used in transfection-based systems to generate recombinant antibodies, including neutralizing antibodies against papillomavirus genotypes [83] and antibodies targeting the hemagglutinin of highly pathogenic avian influenza virus H5N1 [84]. Furthermore, a single-chain variable fragment (scFv) derived from the 5G2 hybridoma, which produces IgM antibodies against REST, has been developed for the clinical evaluation of REST as a potential biomarker of precancerous lesions and cervical squamous cell carcinoma [18,85].

Hybridoma technology is not limited to producing monoclonal antibodies. T-cell hybridomas have emerged as valuable research tools. These systems enable the investigation of T-cell biology, including the characterization of atypical CD4^+^CD8^+^ populations observed in SATB1-deficient mice with Sjögren’s syndrome [86]. Moreover, T-cell hybridomas have been used as highly sensitive biosensors to measure the functional activity of endoplasmic reticulum aminopeptidases 1 and 2 [87].

Furthermore, hybridoma cell lines are considered a promising source of extracellular vesicles with defined immunological characteristics. For instance, the OKT3 hybridoma, which produces murine IgG specific to human CD3, secretes exosomes displaying these antibodies on its surface. These vesicles trigger strong T-cell cytokine responses and demonstrate increased stability compared to soluble antibodies [88].

Hybridoma cells represent a useful model for evaluating the cytotoxicity and biological activity of various compounds. Using the BAP3 hybridoma cell line, which produces murine IgG specific to human trophoblastic β-1 glycoprotein, the effects of graphene oxide nanoparticles functionalized with linear and branched polyethylene glycol were investigated. Graphene oxide nanoparticles at a concentration of 25 μg/mL decreased cell viability, with cytotoxicity diminishing as particle size increased and correlating with nanoparticle internalization. Due to their high proliferative capacity and plasma cell–like characteristics, hybridomas serve as a relevant model for evaluating the effects of different substances on antibody-producing cells [89]. Furthermore, B-cell hybridoma cells (line 53-6.7 from ATCC) were used to assess the biocompatibility of gadolinium oxide (Gd_2_O_3_:Er^3+^, Yb^3+^) nanostructures. Following incubation with nanoparticles and nanorods (concentrations up to 100 μg/mL for 48 h), the hybridoma cells consistently showed high viability and proliferation, with no morphology- or size-dependent toxic effects [90]. Although hybridomas cannot replace primary cultures, they provide a reproducible preliminary platform for screening nanomaterial effects on antibody-secreting cells.

Hybridoma cells have been used as a model system to study the proliferative effects of β-lactoglobulin (LG). It was shown that LG at a concentration of 5 mg/mL stimulates hybridoma proliferation through interaction with membrane-bound IgM, which was identified as the LG receptor. These findings indicate that hybridomas can serve as a convenient cellular model for investigating receptor-mediated effects of proteins on cell proliferation [91].

Hybridoma technology remains a versatile platform for monoclonal antibody generation, with broad applications in diagnostics, fundamental research, and therapeutic development. Furthermore, hybridoma cell lines are used as a source of genetic material for antibody engineering, as model systems for cytotoxicity evaluation, and as producers of extracellular vesicles with specific immunological characteristics.

### 2.4. Challenges in Hybridoma Technology

Despite its revolutionary impact on biomedicine and biotechnology, hybridoma technology faces a series of fundamental challenges that compromise the reliability, reproducibility, and long-term utility of monoclonal antibodies produced by this method. These limitations have become increasingly important as monoclonal antibodies constitute one of the most widely used research reagents. Indeed, antibodies have been recognized as one of the major contributors to the ongoing reproducibility crisis in biomedical research [92,93].

One of the most significant limitations of hybridoma technology is the genetic instability of hybridoma cell lines during prolonged cultivation [94,95]. Hybridomas are inherently chromosomally unstable due to their origin as fusion products between antibody-producing B lymphocytes and myeloma cells. Therefore, the loss of antibody productivity is a reproducible biological phenomenon associated with progressive genetic alterations during prolonged cultivation [95]. These genetic alterations include chromosomal rearrangements, gene loss, and genetic drift [94,96].

Another critical issue is the assumption that hybridoma-derived antibodies are always truly monoclonal. A comprehensive study analyzing antibody mRNA from 185 randomly selected hybridoma clones revealed that 31.9% of hybridomas producing “monoclonal” antibodies actually secrete multiple antibody species [97]. These hybridomas express one or more additional functional heavy and light chains. These extra chains frequently form unintended antibody species with altered affinity, decreased specificity, and increased nonspecific binding, thereby compromising experimental reproducibility [97,98]. The study also demonstrated that the most abundant antibody transcripts within a hybridoma do not necessarily encode the antigen-specific antibody, highlighting the limitations of conventional cloning and the need for molecular validation.

Thus, sequencing of immunoglobulin variable-region genes [99] has become the most reliable approach for confirming the true monoclonality of hybridoma cells [97,100]. Moreover, given the inherent instability of hybridoma cell lines and the risk of irreversible loss caused by contamination, freezer failure, genetic drift, or declining viability—hybridoma sequencing secures the antibody for the long term [100]. Without proper preservation, the unique genetic sequence encoding a valuable antibody may be lost forever, making it impossible to regenerate or reproduce the antibody in the future. Once the antibody genes are identified, they provide a permanent digital record that can be archived indefinitely and reproduced independently of the original hybridoma.

The modern trend is to sequence antibody genes from hybridomas and transfer them into recombinant expression systems [100]. This strategy preserves the unique binding properties of the original clone while conferring all the advantages of recombinant antibodies: genetic stability, batch-to-batch reproducibility, and a defined sequence [101]. The future of hybridoma technology is therefore closely linked to recombinant antibody technologies rather than representing a competing platform. An emerging workflow involves generating antibodies by conventional hybridoma methods, sequencing the paired variable heavy and light chain genes, and subsequently expressing the recombinant antibody in mammalian systems, e.g., CHO and HEK293 cells [93,100].

## 3. Functional Parameters of Hybridoma Cells

When working with hybridoma cells, it is standard practice to evaluate three key functional parameters: viability, proliferative activity, and productivity [102]. In addition to proliferation analysis, the evaluation of apoptosis is critically important. The high proliferative activity and viability of hybridoma cells are determined by genes derived from the tumor cell, whereas the ability to synthesize antibodies is conferred by genes originating from the mature B cell. A comprehensive evaluation of these three parameters not only characterizes the biological activity of the compounds under investigation but also allows researchers to distinguish substances based on their differential effects on cellular functional parameters. This approach is particularly important because an increase in one parameter may be followed by a decrease in another, necessitating a balanced interpretation of the results. Moreover, a comparison of the growth kinetics of producing and nonproducing hybridoma cells in batch culture shows that the latter exhibit a higher specific growth rate [103].

The complexity of this balance is especially pronounced during the initial generation of hybridomas. Immediately after cell fusion and during primary outgrowth, non-producers or low-producers often outcompete high-producing clones because of their superior proliferation rates, creating a pronounced selective disadvantage for the desired cells [50].

A wide range of methods is available for evaluating cell proliferation, viability, apoptosis, and productivity. This review primarily presents those most commonly applied to hybridoma cell lines.

### 3.1. Viability

**Figure 3:**
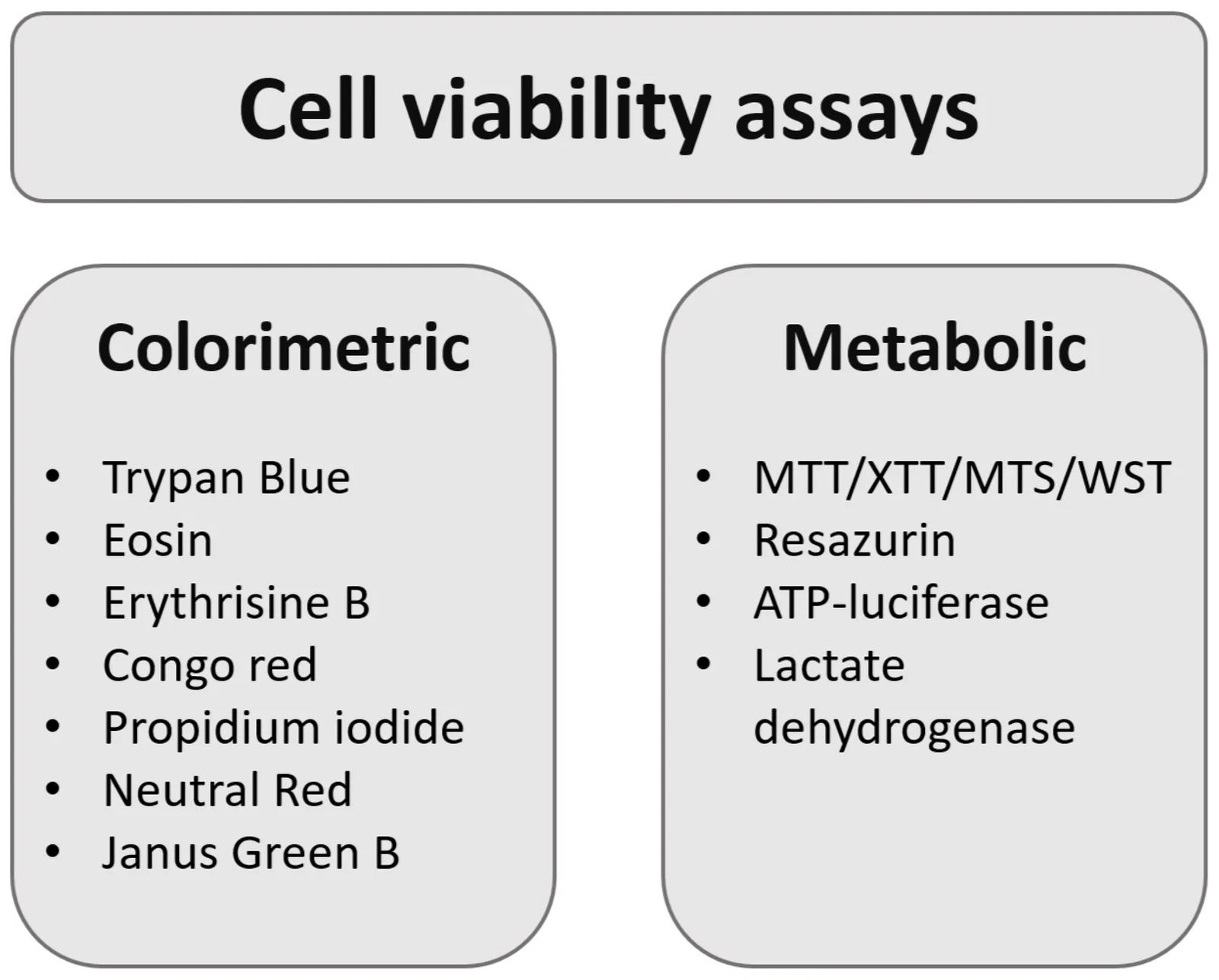
Common assays for cell viability determination.

Cell viability is defined as a quantitative measure of the number of cells that retain functional activity [104]. The methods used for its evaluation, presented in Figure 3, are conventionally classified as colorimetric and metabolic activity-based assays.

Colorimetric methods are among the most accessible and widely applicable ones for hybridoma cells [89,90,105,106]. These include dye exclusion and dye uptake assays [4]. Dye exclusion assays are based on the principle that dead cells lose membrane integrity, allowing dyes to penetrate the cell [107]. The most commonly used dyes include trypan blue, eosin, erythrosine B, Congo red, and propidium iodide [4,107,108]. Cell viability is calculated as the ratio of unstained cells to the total cell number. A modern approach involves the use of Zombie Aqua dye, which provides high-accuracy determination of hybridoma cell viability using flow cytometry and fluorescence microscopy [89].

Dye uptake methods are based on the ability of viable cells to accumulate dyes such as neutral red and Janus Green B [4]. The former is retained within lysosomes due to the pH gradient, whereas the latter is selectively taken up by active mitochondria and reduced by enzymes of the respiratory chain to form a colored product. The amount of dye accumulated is quantified spectrophotometrically [4].

Comparative studies have shown that the differences between trypan blue staining and neutral red staining [109], as well as between trypan blue and fluorescein diacetate (FDA) and propidium iodide (PI) [110], are minimal in the context of hybridoma cell lines. This allows any of these methods to be used in routine practice. However, another study has highlighted limitations associated with the use of trypan blue. When comparing viable cell counts obtained using trypan blue with those obtained by dual fluorescent staining with FDA and PI, it was found that, as hybridoma cultures age, trypan blue may significantly overestimate viability. This results in an underestimation of the proportion of dead cells and an inaccurate assessment of the overall culture status [111]. These findings underscore the need to validate results using additional methods, particularly when analyzing long-term cultivated cell lines.

Metabolic assays can be interpreted in two ways. On the one hand, the level of metabolic activity correlates with the number of viable cells, as only metabolically active cells are capable of reducing substrates. In this context, these methods are regarded as indirect assays of hybridoma cell viability [19,112]. On the other hand, in experiments involving actively proliferating hybridoma cultures, changes in the metabolic signal are frequently used as an indicators of proliferation [13,91,113]. This approach is based on the assumption that an increase in signal reflects not only the maintenance of viability but also an increase in cell number within the population. However, such an interpretation requires caution, as metabolic activity per cell may vary depending on the physiological state, culture conditions, and cell cycle stage.

Thus, metabolic assays cannot always be unambiguously assigned to a single category of functional analyses. Within the scope of this review, we consider them primarily as methods for assessing cell viability.

Metabolic assays are based on measuring enzyme activity or the concentration of metabolites in viable cells. Tetrazolium salts are compounds that, upon reduction, are converted into intensely colored formazans [4,107]. This reduction occurs via mitochondrial enzymes in metabolically active cells [3,4,107,114]. Common tetrazolium salts include MTT, XTT, MTS, and WST, an analog of MTS [3,4,107,114,115]. Absorbance is measured spectrophotometrically at 540 nm for MTT (blue formazan), 450 nm for XTT (orange formazan), and 490–500 nm for MTS (red-orange formazan) [4,107,114].

Resazurin, also known commercially as Alamar Blue, is a non-toxic, cell-permeable dye that is blue in its oxidized form and pink in its reduced form. The oxidized form, resazurin, is reduced by mitochondrial enzymes in viable cells to resorufin. Fluorescence is measured at wavelengths of 570 nm and 630 nm, and the amount of reduced dye correlates with the number of viable cells [4,107].

Intracellular ATP is a reliable indicator of cell viability [107]. ATP concentration is commonly determined using a luciferase-based luminescence assay because ATP is required for the luciferase-catalyzed conversion of luciferin [4,107].

Lactate dehydrogenase (LDH) is a cytoplasmic enzyme that is released upon membrane damage [4,107,108]. Its concentration in the supernatant correlates with the number of dead cells [19]. The enzyme catalyzes the reversible conversion of lactate to pyruvate, coupled with the reduction in NAD^+^ to NADH and vice versa [4,107,108]. The standard approach involves monitoring the change in NADH absorbance at 340 nm, followed by calculation using an appropriate formula [116].

### 3.2. Proliferation

Hybridoma cell proliferation proceeds according to the typical mammalian cell cycle. This process consists of four phases: G1 (cell growth and preparation for DNA synthesis), S (DNA replication), G2 (preparation for cell division), and M (mitosis) [117,118,119].

The transition from the G1 phase to the S phase is a critical event that determines subsequent cell proliferation and viability [120]. Studies of antibody synthesis have shown that per-cell productivity is highest during the G1 and G2 phases, whereas it decreases during mitosis and is lowest in the S phase [121]. At the same time, a positive correlation has been observed between overall culture productivity and the fraction of cells in S phase [122].

Under standard culture conditions, hybridoma cells have a doubling time of approximately 1–2 days [123]. Proliferation rates are strongly influenced by culture conditions. One of the limiting factors is glutamine; its exhaustion leads to a reduction in cell growth [124]. Cytokines play a crucial role in regulating hybridoma growth and survival. For many murine hybridomas, interleukin-6 (IL-6) is a key factor. Binding of IL-6 to its receptor activates the Ras/MAPK and PI3K/Akt signaling pathways, promoting the transition from G1 to S phase and supporting cell viability [120,125]. Disruption of these signaling pathways can result in cell cycle arrest and apoptosis induction. For example, IL-6 deprivation leads to G1-phase arrest in hybridoma cells and prevents the accumulation of S-phase cyclins, ultimately resulting in cell death [126]. Similar effects are observed when the cell cycle is arrested by thymidine or by serum starvation [120].

Comprehensive characterization of a cell culture requires the simultaneous evaluation of proliferation, viability, and cell death [127].

Numerous approaches exist for evaluating cell proliferation. The simplest and most cost-effective method is cell counting using a hemocytometer or its analogs in combination with light microscopy [128,129]. However, this method provides only approximate cell number dynamics, is labor-intensive, and loses accuracy at high cell suspension densities [128].

**Figure 4:**
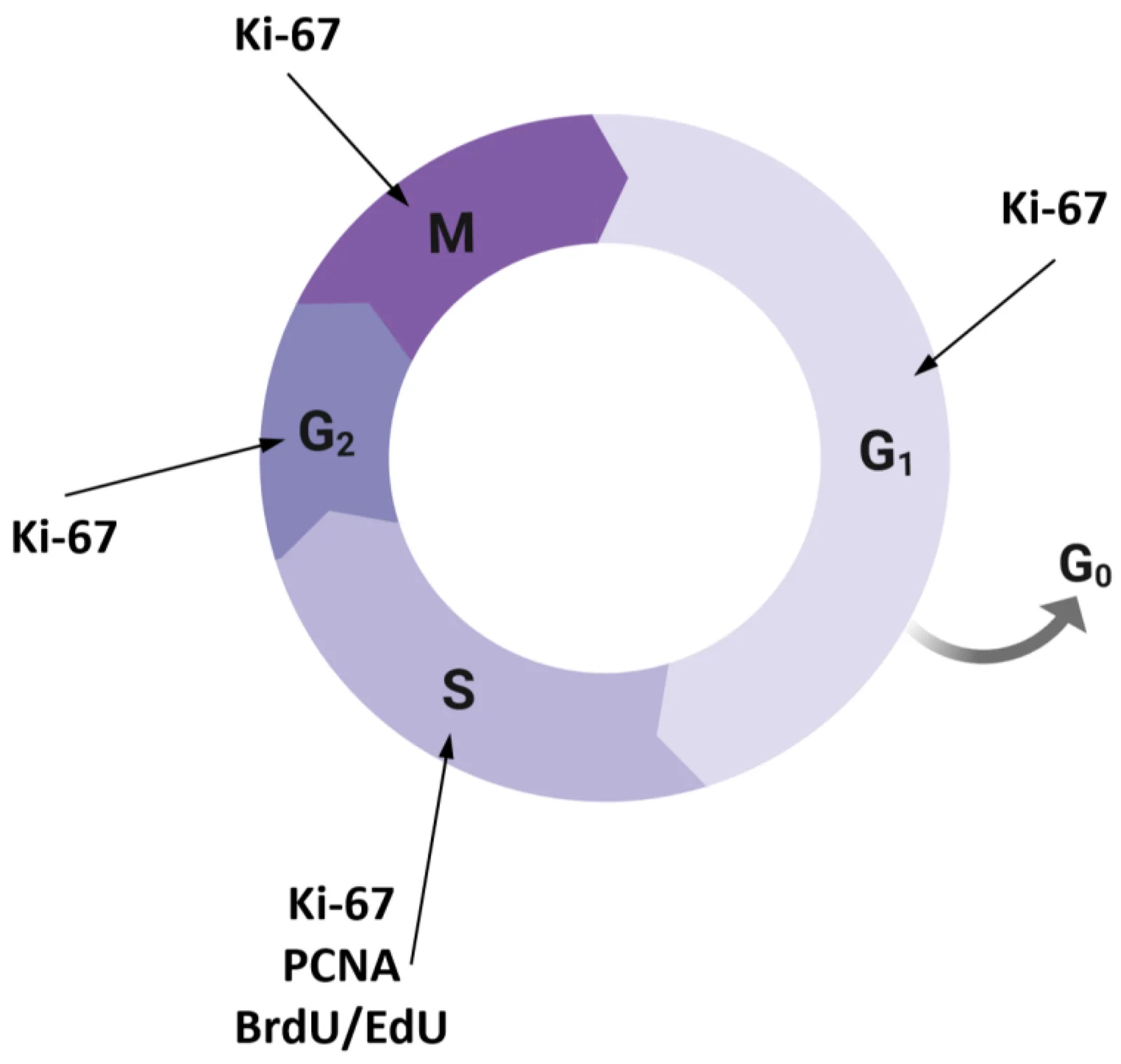
Cell cycle phases and associated molecular markers (created with BioRender.com). G1—cell growth and preparation for DNA synthesis; S—DNA replication; G2—preparation for cell division; M—mitosis; G0—quiescent state.

A more informative approach is the analysis of molecular proliferation markers, as shown in Figure 4. The nuclear protein Ki-67 is expressed during all phases of the cell cycle except G0 phase and therefore allows evaluation of the fraction of actively proliferating cells [130,131]. Synthesis of proliferating cell nuclear antigen (PCNA) begins in late G1 phase and reaches its peak during S phase; it subsequently declines during G2 and is virtually absent during mitosis [3,132,133]. Nucleoside analogs such as BrdU and EdU are added to the culture medium and incorporated into newly synthesized DNA, thereby enabling detection of S-phase cells [3,118,134].

An alternative approach to assessing cell cycle status is quantitative measurement of intracellular RNA using dual staining with Hoechst 33342 and Pyronin Y, as actively proliferating cells typically contain higher RNA levels than quiescent cells [135].

Hybridoma cell proliferation can also be evaluated using the carboxyfluorescein succinimidyl ester (CFSE) dilution assay [90]. The CFSE method relies on the principle of fluorescent dye dilution upon cell division. After binding to intracellular proteins, CFSE is equally distributed between daughter cells during symmetric mitosis, resulting in a stepwise twofold decrease in fluorescence intensity with each generation, which can be measured by flow cytometry [136].

Hybridoma cells exhibit high sensitivity to stress [137]; therefore, the assessment of apoptosis is critically important. Apoptosis is a form of programmed cell death. In most cases, cell death proceeds via the mitochondrial apoptotic pathway, followed by caspase activation [126].

One approach to enhancing cellular robustness is overexpression of anti-apoptotic proteins of the Bcl-2 family [138,139]. In Sp2/0 hybridoma cells, this modification approximately doubles the duration of the productive phase and increases antibody production by about 40% [138]. Similarly, overexpression of Bcl-2 increases the maximum viable cell density by 45%, delays the onset of apoptosis, and prolongs culture viability [139]. Pharmacological inhibition of apoptosis can also enhance cell survival; for example, the pan-caspase inhibitors z-VAD-fmk and Ac-DEVD-cho block effector caspases and maintain hybridoma viability under glutamine deprivation for up to 36 h [140].

Apoptosis in hybridoma cells is commonly detected using Annexin V [141], a marker that binds to phosphatidylserine exposed on the outer surface of the cell membrane during the early stages of apoptosis [4,142]. The combined use of Annexin V and PI allows discrimination between early apoptosis (Annexin V^+^/PI^−^) and late apoptosis or necrosis (Annexin V^+^/PI^+^) [4]. In addition, the TUNEL assay (terminal deoxynucleotidyl transferase dUTP nick end labeling) is used to assess apoptosis in hybridoma cells [143]. This method is based on the use of the enzyme terminal deoxynucleotidyl transferase (TdT), which catalyzes the incorporation of labeled deoxynucleotides into free 3′-OH termini of DNA, thereby enabling detection of DNA fragmentation—one of the hallmarks of apoptosis [144,145]. The DNA laddering assay is also used to detect apoptosis in hybridoma cells [146]. This method is based on the identification of a characteristic DNA fragmentation pattern by agarose gel electrophoresis [4].

### 3.3. Productivity

Productivity represents the capacity of a cell to carry out its specialized function, namely the synthesis and secretion of the target protein. At the molecular level, it is driven by processes associated with expression of the gene encoding the target protein—in hybridomas, the immunoglobulin light- and heavy-chain genes [147]—as well as by post-translational modifications and protein synthesis [148]. A detailed discussion of the molecular mechanisms regulating productivity is beyond the scope of this review.

In the context of hybridoma productivity, it is useful to differentiate parameters such as target product titer and specific productivity. The target product titer represents the final concentration of antibodies in the culture medium. This parameter is equivalent to overall cellular productivity and is typically expressed as antibody mass per unit volume. Mathematical models describing target product titer have been reported in several studies [149,150]:

(1) $$Titer=Q_{p}\times \;\int X_{v}dt$$

where Titer is target product titer, $Q_{p}$ is specific productivity, $X_{v}$ is viable cell density over the cultivation period.

Specific productivity is generally defined as the amount of target product produced by a single cell per unit time. This parameter depends on both the antibody concentration in the medium and the viable cell count. Accordingly, evaluation of specific productivity requires consideration of both the dynamics of viable cell density and the final antibody concentration in the medium (titer). The mathematical formulation can be derived from the model described above [149,150]:

(2) $$Q_{p}=\frac{Titer}{\int X_{v}dt}\;$$

where $Q_{p}$ is specific productivity, Titer is target product titer, $X_{v}$ is viable cell density over the cultivation period.

Enzyme-linked immunosorbent assay (ELISA) and dot-immunoblotting are commonly used to assess the productivity of hybridoma cell lines. Among these methods, ELISA is the preferred [13,151,152,153], primarily due to its high sensitivity, specificity, and quantitative capacity.

Enzyme-linked immunosorbent assay (ELISA) is a versatile immunological method widely used in fundamental and applied research, as well as in clinical practice and diagnostics. The method relies on the specific interaction between an antigen and a primary antibody targeting the analyte of interest. Detection of the antigen–antibody complex is typically achieved using enzyme-conjugated antibodies that catalyze a reaction with an appropriate substrate. The reaction products are quantified using either a luminometer or a spectrophotometer. Depending on the experimental design, ELISA can be classified into direct, indirect, sandwich, and competitive formats, which differ in reagent types, step sequences, and assay conditions [154].

An alternative approach is dot-immunoblotting, which is used for the rapid screening of hybridoma clones and evaluation of their secretory activity [155,156]. In the classical assay format, the nitrocellulose membrane is precoated with affinity-purified anti-mouse immunoglobulins (derived from goat or rabbit), followed by incubation with the hybridoma culture supernatant. Secreted monoclonal antibodies are captured on the membrane surface and subsequently detected using enzyme-labeled reagents, most commonly horseradish peroxidase, which enables signal visualization and comparative assessment of antibody production levels [157].

## 4. Differential Modulation of Hybridoma Cells Functional Parameters

Various strategies have been developed to enhance target product titers in hybridoma cells, including optimization of culture conditions [158], fed-batch cultivation [159], vitamin supplementation [160], use of antioxidants such as coenzyme Q10 [161], and use of various chemical compounds [6]. In these cases, productivity is enhanced either through an increase in viable cell numbers [162] or through an increase in specific productivity [163]. However, proliferation alone does not always correlate with increased antibody production, which underscores the importance of identifying compounds that stimulate cell growth without reducing specific productivity.

The studies summarized in Table 2 investigate the correlation between proliferation and specific productivity. According to the proposed model, reducing proliferation to a certain level increases specific cell productivity; however, overly suppressed growth also decreases productivity [6]. This finding supports the concept of intracellular resource reallocation, wherein artificial suppression of cell division may redirect metabolic resources toward antibody synthesis and secretion. It has been demonstrated that an increase in specific growth rate results in a reduction in the specific production rates of both ATP and antibodies [164]. The corresponding mathematical model describes the dependence of productivity on the proliferation rate through the regulation of transcription and translation at different stages of the cell cycle [6]. The experimental data below further show that inhibiting proliferation can indeed increase cell-specific productivity. Growth suppression may be induced either by unfavorable culture conditions or by compounds with growth-inhibitory activity.

Osmolarity, defined as the total concentration of osmotically active particles per unit volume, determines the direction of water flux across the cell membrane and represents a key physicochemical parameter governing the balance between cell proliferation and protein synthesis. The use of culture media with elevated osmolarity can enhance hybridoma productivity [165,166]. However, this is typically followed by a decrease in proliferation rate and an increase in cell volume. Effective application of hyperosmotic conditions often requires prior adaptation of the cells. The physiological osmolarity of the medium is approximately 250–300 mOsm. Sodium chloride is commonly used to increase it. Increasing osmolarity to 435 mOsm enhances specific productivity by approximately 2.2-fold but does not improve overall productivity because of reduced cell growth [165]. A combination of moderate osmolarity elevation (350 mOsm) with sodium butyrate (0.1 mM) resulted in a twofold increase in antibody titer at the end of cultivation [166]. Despite its relative simplicity, this strategy requires prolonged adaptation [166] and careful, cell line–specific optimization [175].

A wide range of compounds has been reported as growth inhibitors of hybridoma cells. Among the most extensively studied is sodium butyrate [167,168], commonly applied at a concentration of 1 mM. Notably, sodium butyrate is effective not only in hybridoma systems but also in CHO cell cultures [168]. Colchicine has likewise been proposed as a promising agent for enhancing hybridoma productivity. The addition of colchicine at a concentration of 10 ng/mL to a medium supplemented with 1 mM sodium butyrate resulted in a 43% increase in productivity [169]. The use of colchicine represents an effective strategy for increasing monoclonal antibody yield, particularly in hybridoma lines that respond poorly to conventional productivity enhancers. This effect is related to the ability of colchicine, a microtubule and mitotic inhibitor, to slow cell proliferation [176].

Similar effects have been reported for other regulators of the cell cycle. For example, caffeine can induce cell cycle arrest in the G0/G1 phase [177]. At a concentration of 1.92 mM, caffeine increased hybridoma-specific productivity by 2.8-fold [163] and 1.8-fold [170], although no corresponding increase in final antibody titer was observed. Bacterial lipopolysaccharide (LPS) at a concentration of 100 pg per cell has also been reported to suppress proliferation while increasing overall hybridoma productivity by up to 2.5-fold [171]. However, its practical application is limited by challenges associated with downstream purification. Similarly, low concentrations of DMSO (0.2%) can enhance hybridoma productivity by approximately twofold [172], likely through induction of cell cycle arrest in the G1 phase [178]. Notably, DMSO at low concentrations does not affect monoclonal antibody glycosylation, highlighting its potential for industrial-scale applications [172].

Polyamines are also considered potential regulators capable of enhancing hybridoma productivity and modulating their functional activity. Studies using the human hybridoma cell line HB4C5 have demonstrated that certain polyamines significantly enhance IgM production under serum-free conditions [173,174]. For example, the addition of spermine at a concentration of 7.3 mM and spermidine at 4.5 mM increased IgM production approximately sixfold [173,174]. In both cases, the increase in specific productivity was followed by growth inhibition. Thermine at a concentration of 2 mM also exhibited a pronounced stimulatory effect, increasing IgM production by approximately 5.3-fold without noticeably suppressing cell proliferation [174]. Although the final antibody titer was not reported, it can be assumed that it increases due to enhanced specific productivity while maintaining proliferative activity. In contrast, triethylenetetramine induced only a transient increase in IgM production and exhibited marked cytotoxicity, limiting its applicability in long-term culture [174].

In addition to small molecules, cytokines also play a significant role in regulating hybridoma productivity. For example, recombinant human IL-6 at a concentration of 2 ng/mL increased the specific productivity of hybridomas approximately fivefold in serum-free medium [151]. Furthermore, IL-6 can inhibit apoptosis in hybridoma cells, thereby extending culture duration and increasing overall antibody yield [179].

One should note that inhibition of proliferation combined with stimulation of specific productivity does not always result in a higher antibody titer. This effect can be explained by a substantial reduction in viable cell numbers, which may ultimately lead to lower target product titers. At the same time, it has been demonstrated that the addition of growth inhibitors at high cell densities can increase the yield of the target product by up to twofold [167]. Growth inhibitors that disrupt antibody synthesis reduce both the specific productivity and the target product titer [6].

Thus, optimization of hybridoma cells functional parameters in established, stable cell lines requires a strict balance between proliferation regulation and product yield enhancement, making the identification of reliable productivity-enhancing agents a promising and important area of research.

## 5. Conclusions

Hybridoma cell lines represent a unique biotechnological model that combines unlimited proliferative capacity with the ability to produce highly specific antibodies. This dual nature underlies their significance not only as a classical tool for generating monoclonal antibodies but also as a versatile platform for comprehensive evaluation of the biological activity of various compounds.

Compared with conventional cell-based assays, hybridoma cell lines enable the simultaneous evaluation of proliferation, viability, and productivity—the last of which represents a specific functional parameter. Since none of these parameters alone can fully reflect the functional state of the culture, their integrated evaluation offers the most accurate insight into the cellular response to the investigated compounds.

Importantly, these parameters are connected through a complex functional interplay. An increase in specific productivity may correlate with cell cycle inhibition, whereas stimulation of proliferation can lead to a reduction in productivity. Such differential modulation effects indicate cellular resource reallocation and underscore the need for comprehensive analysis of functional parameters. Accordingly, evaluation of hybridoma cells functional parameters enables the detection and characterization of these effects. Thus, hybridoma cells should be considered not only a production platform but also a highly informative biological model for both fundamental and applied research.

Nevertheless, hybridoma technology remains a relevant platform for the generation of monoclonal antibody-producing cells, making the identification of productivity-enhancing factors a key strategy for optimizing the biotechnological production of target proteins.

## Figures and Tables

**Table 1:** Advances in Hybridoma Technology.

No	Hybridoma Technology Stage	Optimization Strategy	Description of Improvement	Ref.
1	Fusion	PEG pre-incubation	Pre-incubation of myeloma cells and lymphocytes with 0.25% PEG for 90 min at 37 °C	[29]
2	Fusion	Electrofusion pulse optimization	Use of nanosecond and microsecond pulses with low peak power in electrofusion	[30]
3	Fusion	Fluorescence-activated cell sorting enrichment prior to fusion	Sorting of B-cells by FACS before electrofusion	[21]
4	Fusion	Microfluidic electrofusion chip	Electrofusion in microfluidic droplet-based platform	[31]
5	Post-fusion Survival	Macrophage-conditioned medium	Addition of macrophage-conditioned medium	[32]
6	Post-fusion Survival	Thymocyte-conditioned medium	Replacement of feeder cells	[29]
7	Selection	FACS-based screening	Antigen-specific sorting using fluorescence labeling	[33,34,35,36]
8	Selection	Membrane Ig-based screening (MIHS)	Detection via B-cell receptor antigen binding	[37]
9	Selection	Magnetic particle isolation	Antigen-coated magnetic particles	[38]
10	Selection	Semisolid media	Spatial isolation of hybridoma colonies	[39,40]
11	Selection	ClonePix 2	Automated colony screening	[41,42]
12	Selection	Microwell chip with antibody capture membrane	Self-seeding chip capturing secreted antibodies	[43]
13	Selection	Surface plasmon resonance	Kinetic characterization of antibody–antigen interaction	[44,45,46,47]
14	Selection	Microarray-based screening	Antibody-immobilized microarray on Cys-Protein G-coated epoxy slides with parallel noncompetitive and competitive monovalent fluorescent tracer incubation.	[48]

**Table 2:** Strategies for enhancing hybridoma cell productivity. ↑—increase, ↓—decrease, =—no effect, N/S—not stated in the article.

No	Cell Treatment	Effect on Proliferation	Effect on Specific Productivity	Effect on Antibody Titer	Ref.
1	Increase in osmolarity	↓	↑	=	[165]
2	Increase in osmolarity + Sodium butyrate addition	↓	↑	↑	[166]
3	Sodium butyrate addition	↓	↑	↑	[167,168]
4	Colchicine addition	↓	↑	↑	[169]
5	Caffeine addition	↓	↑	N/S	[163,170]
6	LPS addition	↓	↑	↑	[171]
7	DMSO addition	↓	↑	N/S	[172]
8	Spermine addition	↓	↑	N/S	[173]
9	Spermidine addition	↓	↑	N/S	[174]
10	Thermine addition	=	↑	N/S	[174]
11	Triethylenetetraamine addition	↓	↑	N/S	[174]
12	IL-6 addition	↑	↑	N/S	[151]

## Data Availability

No new data were created or analyzed in this study. Data sharing is not applicable to this article.

## Abbreviations

The following abbreviations are used in this manuscript:

ATCC	American Type Culture Collection
BrdU	5-bromo-2′-deoxyuridine
CALR	Calreticulin
CFSE	Carboxyfluorescein succinimidyl ester
EdU	5-ethynyl-2′-deoxyuridine
ELISA	Enzyme-linked immunosorbent assay
FACS	Fluorescence-activated cell sorting
FDA	Fluorescein diacetate
HAT	Hypoxanthine, aminopterin, thymidine
HGPRT	Hypoxanthine-guanine phosphoribosyltransferase
IL-6	Interleukin-6
LDH	Lactate dehydrogenase
LG	β-lactoglobulin
LPS	Lipopolysaccharide
MIHS	Membrane Ig-based screening
MTS	3-(4,5-dimethylthiazol-2-yl)-5-(3-carboxymethoxyphenyl)-2-(4-sulfophenyl)-2H-tetrazolium
MTT	3-(4,5-dimethylthiazol-2-yl)-2,5-diphenyltetrazolium bromide
PCNA	proliferating cell nuclear antigen
PEG	Polyethylene glycol
PI	Propidium iodide
scFv	Single-chain variable fragment
SPR	Surface plasmon resonance
TdT	Terminal deoxynucleotidyl transferase
TUNEL	Terminal deoxynucleotidyl transferase dUTP nick end labeling
VEGFA	Vascular endothelial growth factor A
XTT	2,3-bis(2-methoxy-4-nitro-5-sulfophenyl)-5-[(phenylamino)carbonyl]-2H-tetrazolium hydroxide
